# Influence of Scenting Time on the Volatile Compounds and Sensory Attributes of Jasmine Yellow Tea

**DOI:** 10.3390/foods15101712

**Published:** 2026-05-13

**Authors:** Jiaqi Ying, Youcang Jiang, Huimin An, Yuan Chen, Jiashun Liu, Yiwen Huang, Sirui Wang, Wei Wang, Shi Li, Zhonghua Liu, Jianan Huang

**Affiliations:** 1 Key Laboratory of Tea Science of Ministry of Education, Hunan Agricultural University, Changsha 410128, China; 15179871658@163.com (J.Y.);; 2 National Research Center of Engineering and Technology for Utilization of Botanical Functional Ingredients, Hunan Agricultural University, Changsha 410128, China; 3 China Tea (Hunan) Co., Ltd., Changsha 410299, China; 4 Yuelushan Laboratory, Changsha 410128, China; 5 National Key Laboratory for Tea Plant Germplasm Innovation and Resource Utilization, Hunan Agricultural University, Changsha 410128, China; 6 Key Laboratory for Evaluation and Utilization of Gene Resources of Horticultural Crops, Ministry of Agriculture and Rural Affairs of China, Hunan Agricultural University, Changsha 410128, China

**Keywords:** jasmine yellow tea, scenting time, volatile compounds, aroma quality

## Abstract

Scenting time is a critical parameter that not only shapes the aromatic characteristics of jasmine tea but also influences its processing strategies. This study aims to elucidate how scenting time influences the aroma quality of jasmine yellow tea (JYT) through the integration of sensory evaluation with volatile compound profiling and the use of multivariate analyses, including principal component analysis (PCA) and orthogonal partial least squares–discriminant analysis (OPLS-DA), alongside complementary analytical approaches. Results demonstrated that the optimal sensory aroma quality of JYT was achieved when the scenting duration ranged from 8 to 12 h. Three hundred volatile compounds were detected, fourteen of which were identified as key contributors to JYT’s distinctive aroma profile. These include indole, methyl anthranilate, methyl salicylate, and *δ*-Cadinene, all of which exerted a significant influence on the overall aroma quality score. The identification of these principal volatile components provides valuable insights for optimizing JYT manufacturing processes and offers a theoretical basis for establishing systematic aroma quality assessment protocols.

## 1. Introduction

Yellow tea is recognized as one of the six major categories of tea in China and is classified into yellow bud tea, large yellow tea and small yellow tea based on the picking standards applied to the raw materials [1]. Its characteristic yellowing process gives rise to the “three yellows” attribute of yellow tea—yellow dry tea, yellow infusion, and yellow tea dregs [2]. The fundamental distinction between yellow tea and green tea lies in the “yellowing” process. This crucial “yellowing” step promotes mild non-enzymatic oxidation and limited microbial activity, which can lead to the partial hydrolysis of bitter catechins and the formation of new volatile and non-volatile compounds [3]. As a result, yellow tea typically develops a characteristic aroma profile described as mellow, sweet, and “corn-sweet” or “bakery-like” [4], which is clearly distinct from the fresh, grassy, vegetal, and sometimes chestnut-like notes associated with high-quality green tea [5]. In addition to its distinct sensory qualities, yellow tea has been associated with a range of health benefits, including blood sugar regulation, antioxidant activity, hepatoprotective effects, and modulation of intestinal flora [6,7,8,9]. According to statistics from the China Tea Circulation Association, yellow tea production in 2023 accounted for 0.69% of the national total, with an output value of USD 431 million [10]. Its steady rise in consumption suggests significant market potential.

The main characteristics of Jasmine fresh flowers are sweetness, freshness, and jasmine fragrance [11]. This distinctive aroma results from the combined action of a range of volatile constituents, including floral-scented terpenoids such as linalool and α-farnesene, the multi-scented indole, and the fruit-scented methyl anthranilate, which are among the most abundant volatiles in jasmine [12]. Jasmine tea is a reprocessed tea produced by scenting a tea base with jasmine flowers and has considerable industrial and commercial value. Current research on jasmine tea predominantly focuses on the flavor quality and process optimization of jasmine green tea. For instance, benzyl acetate is identified as a major aroma constituent responsible for the floral and fruity notes in jasmine green tea, largely due to adsorption from jasmine flowers during scenting [13]. Aroma adsorption is largely determined by the pore volume of the tea matrix, explaining why baked green tea is generally preferred over roasted green tea as a base for jasmine green tea [14]. Moreover, Rahmam et al. [15] reported that treatment with *β*-cyclodextrin enhances the adsorption capacity of tea for aroma compounds. These findings have advanced the optimization of jasmine green tea fragrance. However, the aroma quality and characteristic volatile profile of JYT remain largely unexplored.

Scenting is a key step in establishing the aroma characteristics and overall quality of jasmine tea [16]. Scenting time, in particular, has a profound influence on the retention of floral fragrance and is affected by variables such as temperature and flower quantity [17]. During scenting, continuous respiration of jasmine flowers releases large amounts of carbon dioxide and heat; prolonged scenting may trigger anaerobic respiration, resulting in undesirable off-odors. Conversely, an excessively short scenting time can lead to inadequate floral intensity and poor aroma persistence. Determining the optimal scenting time is therefore essential for balancing aroma quality with process efficiency [18].

In recent years, advancements in machine learning have introduced new approaches to tea quality assessment. For example, a computer vision and color-difference-based method for evaluating jasmine tea quality [19], while an integrated approach combining an electronic nose system, machine vision and decision-making algorithms has been developed to achieve precise classification of jasmine tea grades [20]. These developments highlight the potential for integrating data-driven methods into rapid, objective quality evaluation.

It is hypothesized that an optimal aroma scenting time exists for JYT, which not only enables the effective adsorption of volatile compounds from jasmine but also maintains the integrity of the tea base’s aroma and the jasmine fragrance, while reducing the formation of undesirable odors. In this study, HS-SPME and GC×GC-Q-TOF/MS were employed to characterize volatile compounds in JYT samples. Multivariate statistical techniques, including principal component analysis (PCA), hierarchical cluster analysis (HCA), and orthogonal partial least squares–discriminant analysis (OPLS-DA), were used to identify distinctive volatile markers. Furthermore, partial least squares (PLS) regression was employed to explore the latent relationship and build a correlation model between volatile composition and aroma quality. By determining an optimal scenting time range, this work provides an empirical basis for improving industrial processing protocols, thereby enhancing the quality and market competitiveness of JYT.

## 2. Materials and Methods

### 2.1. Materials

**Figure 1:**
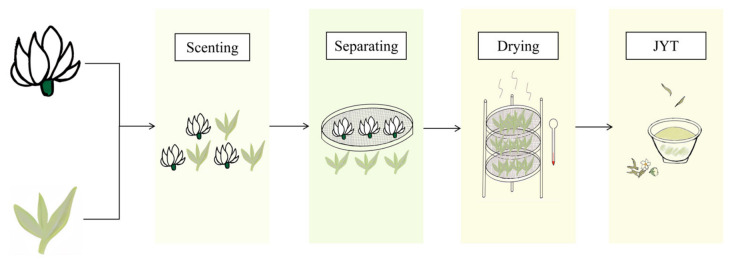
The processing technology of JYT.

The yellow tea base (F0) incorporated in this research originated from Yueyang Suigong Shiba Tea Co., Ltd. (Yueyang, China). Double-petal jasmine (*Jasminum sambac*) blossoms were harvested from Hengzhou City, Nanning, Guangxi Province, China. JYT samples were prepared in collaboration with China Tea (Hunan) Co., Ltd. (Changsha, China) in August 2023, following the processing procedure illustrated in Figure 1. When the jasmine flower buds had opened to approximately 90%, the fresh flowers were mixed with yellow tea to initiate the scenting process. The mixing ratio of jasmine flowers to yellow tea was 3:5, corresponding to samples with 60% of flowers, which ensured sufficient aromatic infusion without overwhelming the intrinsic flavor of the tea base. The blended mixture was then piled to a height of approximately 25–30 cm to facilitate optimal fragrance absorption by the tea leaves. After the scenting process was completed, the spent jasmine flowers were carefully separated from the tea leaves. The scented tea leaves were subsequently dried at 40 °C for 4–5 h, yielding the finished product known as JYT. To investigate the effect of scenting time on aroma development, JYT samples were prepared under six different scenting times, designated as follows: F1 (4 h), F2 (8 h), F3 (10 h), F4 (12 h), F5 (14 h), and F6 (16 h). Upon completion of processing, all samples were placed in sterile polyethylene bags, taken promptly to the laboratory and kept at a temperature of –80 °C until further analysis.

### 2.2. Sensory Evaluation of JYT

**Figure 2:**
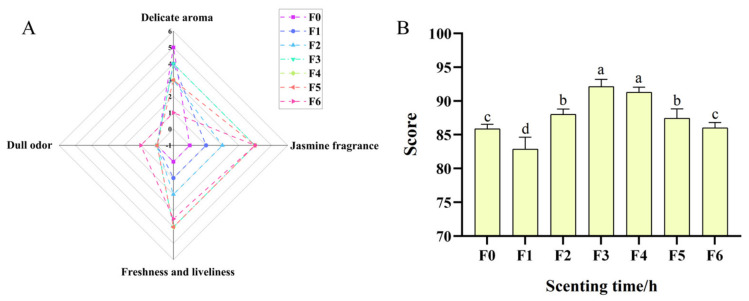
Radar chart of aroma attribute scores (**A**) andSensory evaluation scores of JYT (**B**). F0–F6 represent JYT samples with different scenting time. Lowercase letters (a–d) represent significant differences between groups. Different letters represent significant differences (*p* < 0.05), and there is no significant difference between the columns of the same letter.

Aroma quality assessment of JYT was evaluated in accordance with GB/T 23776-2018 [21]. Seven sensory evaluation experts, each with over 10 years of professional experience in tea sensory assessments, were invited to conduct sensory quality evaluation of JYT in accordance with the relevant national standards (three males and four females). Before the formal evaluation, systematic training was conducted for the seven experts. The training content primarily involves the following: gradient standard calibration training for sensory assessment of scented tea aroma and scented tea aroma sensory evaluation score consistency training. For each evaluation, 3 g of tea were infused in 150 mL of boiling filtered water at 100 °C, with the first steeping lasting 3 min and the second 5 min. Prior to sensory scoring, each tea infusion sample was assigned a unique number and the serving order strictly followed the ascending sequence of these identifiers. The panelists conducted a blind evaluation on each sample independently [22]. Referring to the method described by Ouyang et al. [23], the sensory quality of JYT was assessed by assigning a total aroma score on a 100-point scale, together with ratings for specific sensory attributes. These attributes included freshness, jasmine fragrance, delicate aroma, and dull odor, each evaluated using a 0–5 point intensity scale (where 0 indicates none, 3 represented medium intensity, and 5 indicates very strong). A total of three independent sensory evaluation sessions were conducted under standardized conditions. The mean values obtained from these replicates were used as the representative scores for the fragrance quality of each JYT sample. Evaluation criteria for aroma characterization, along with the corresponding results, are illustrated in Figure 2A,B. Prior to consenting to participate in the study, all subjects were thoroughly informed of the research requirements and potential risks involved. All reviewing experts voluntarily agreed to participate in the sensory evaluation experiments. The participant’s personal information was kept confidential, and sensory data were disclosed only with their explicit consent. The materials utilized in the experiments complied with food quality and safety standards and posed no risk to humans, animals, or the environment. Furthermore, the experimental protocols involving sensory assessments adhered to the relevant operational guidelines established in China.

### 2.3. Volatile Compounds Identification Methods

#### 2.3.1. Extraction of Volatile Components

Tea samples were first thoroughly homogenized and ground to a uniform powder using a grinder. Subsequently, 1.000 g of tea powder was accurately weighed using a ten-thousandth balance in triplicate (*n* = 3) and individually transferred into 20 mL headspace vials. To each vial, 10 μL of ethyl decanoate internal standard solution (50 ppm) and 5.0 mL of boiling water at 100 °C were added, and the vial was immediately sealed. The vials were incubated at 60 °C for 10 min to establish gas–liquid thermal equilibrium prior to headspace analysis.

For headspace solid-phase microextraction (HS-SPME), prior to the adsorption of volatile components from the samples, the 50/30 µm DVB/CAR/PDMS was preconditioned at 250 °C for 30 min in the GC inlet. The 50/30 µm DVB/CAR/PDMS fiber (Supelco, Bellefonte, PA, USA) was then positioned 1 cm above the liquid phase and allowed to adsorb volatiles for 30 min under isothermal conditions (60 °C). The fiber was then desorbed at 250 °C for 10 min at the GC inlet for subsequent analysis.

#### 2.3.2. GC×GC-Q-TOF-MS Analysis Conditions

Volatile compounds were analyzed by GC×GC-Q-TOF-MS. The analytical system consisted of an Agilent 8890 GC-Q-TOF-MS instrument (Agilent Technologies, Santa Clara, CA, USA) equipped with a solid-state thermal modulator (SSM 1820, J&X Technologies, Shanghai, China). The 1-dimensional (1D) and 2-dimensional (2D) chromatographic columns employed were an HP-5MS capillary column (30 m × 250 μm, 0.25 μm) and a DB-17MS capillary column (2.89 m × 180 μm, 0.18 μm), respectively.

Carrier gas and split ratio: Ultrapure nitrogen (purity ≥ 99.999%) was employed as the carrier gas at a constant flow rate of 1.0 mL/min, with a split ratio of 20:1.

Injection and oven temperature program: Gas chromatography was performed with the injector temperature maintained at 250 °C. The oven temperature program was set as follows: initial temperature 40 °C, ramped at 5 °C/min to 180 °C, then further increased at 20 °C/min to 250 °C, and held for 1 min [24].

Mass spectrometry conditions: Mass spectrometry was conducted under the following operating parameters: mass scan range of 45–450 amu, electron ionization energy set at 70 eV, ion source maintained at 200 °C, and transfer line stabilized at 280 °C.

### 2.4. Identification and Quantification of Volatile Components

Qualitative Analysis: Identification followed the protocol of Yin’s method [25]. All data were imported into Canvas Panel 1.0 software for processing with the signal-to-noise ratio set to 10 and the modulation period set to 4. C7-C20 n-alkane was employed for determination of RIs. RI values were calculated according to the equation and then compared with the reported RI data available in the NIST 20 library. If the experimentally determined RI differed from the database RI by no more than 30% (absolute value), the compound was considered to be positively identified as the same compound.

Quantitative Analysis: Volatile compound content was determined using the internal standard method:

*C* = (*R* × 10 μL × 50 mg/L)/*M*

where *C* = concentration of compound (mg/kg); *R* = ratio of compound peak area to internal standard peak area; *M* = sample mass (g).

### 2.5. Odor Activity Values (OAVs) Calculation

OAV was determined by dividing the concentration of a volatile compound by its corresponding odor threshold (OT) in an aqueous medium.

### 2.6. Statistical Analysis

The experiments were carried out in three parallel replicates, and the final results were calculated as the average of these triplicate trials. Several software packages were utilized for data processing, statistical analysis, and graphical representation. Microsoft Office Excel 2021 was used for data organization and numerical calculations. Multivariate statistical analyses, including Principal Component Analysis (PCA), Hierarchical Cluster Analysis (HCA), Orthogonal Projections to Latent Structures Discriminant Analysis (OPLS-DA), and Partial Least Squares (PLS) analysis, were conducted using SIMCA-P 14.1 software. GraphPad Prism 8 was employed to generate line graphs and bar charts, while Origin 2022 was applied for the construction of radar plots. SPSS 27.0.1 was utilized for significance analysis, one-way analysis of variance (ANOVA), and Duncan tests. The OmicStudio online platform (www.omicstudio.cn) was used to produce heatmaps and Upset plots.

## 3. Results and Discussion

### 3.1. Sensory Quality Analysis of JYT with Different Scenting Times

The aroma properties of seven tea samples were evaluated and subsequently categorized into delicate aroma, jasmine fragrance, freshness and dull odor, as shown in Figure 2A. The yellow tea base demonstrated the most pronounced delicate aroma. In contrast, the freshness of the scented tea increased progressively with extended scenting time. However, upon reaching 16 h of scenting, the freshness diminished and developed a dull odor. Following scenting, the overall aroma quality was optimal when scenting lasted between 8 and 12 h (Figure 2B). This trend is consistent with the previous findings [26], which suggested that, within a certain range, increasing scenting time and adding a higher proportion of flowers enhance the fragrance of the tea.

The freshness of the tea increased with scenting time, reaching its maximum value between 10 and 12 h, and then gradually declined. Meanwhile, the intensity of jasmine fragrance increased continuously with longer scenting times, but after 10 h, the jasmine aroma tended to stabilize. The observation implies that when the flower ratio was 60% and the scenting time reached 10 h, the adsorption capacity of yellow tea for jasmine aroma compounds may have reached saturation. Additionally, under such conditions, the capacity of jasmine flowers to emit aromatic volatile substances could potentially be reduced. The overall fragrance quality of jasmine tea relies on the interplay of multiple aroma characteristics and their intensities [27]. Among the samples, F3 achieved the highest aroma score (92), corresponding to the highest freshness and jasmine fragrance (score = 4), whereas F1 obtained the lowest score (83) due to weak jasmine fragrance and poor freshness. The scores of F4–F6 gradually declined, possibly because the activity of jasmine flowers decreased with extended scenting, resulting in slightly unpleasant odors in JYT. These findings indicate that optimized scenting parameters can significantly improve the yellow tea’s aroma quality through effective jasmine aroma integration. Although scenting time is a dominant factor determining the overall quality of JYT, excessive scenting time induces undesirable sensory characteristics, confirming that a longer scenting time does not linearly translate into better aroma quality. Therefore, selecting an appropriate scenting time not only ensures a pleasant aroma but can also reduce production costs.

### 3.2. Dynamic Changes in Volatile Components in JYT During the Scenting Process

**Figure 3:**
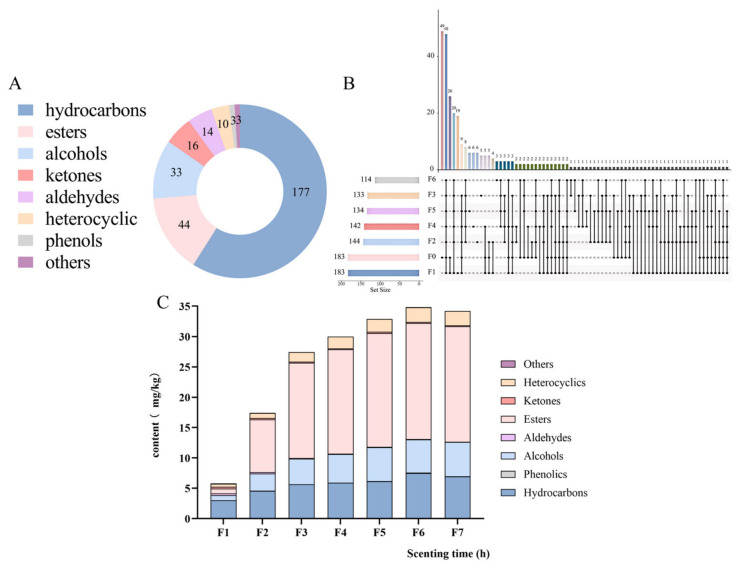
Total volatile compound types and quantities (**A**) in JYT with different scenting times. (**B**) The upset plot of the distribution of volatile compounds in different samples and their content of volatile compounds (**C**).

The volatile profile of JYT is complex and diverse, with 300 volatile components identified, including 177 hydrocarbons (59%), 44 esters (14.67%), 33 alcohols (11%), 16 ketones (5.33%), 14 aldehydes (4.67%), 10 heterocyclic compounds (3.33%), 3 phenols (1%), and 3 others (1%) (Figure 3A). The composition and abundance of detected volatile compounds changed considerably with different scenting times. Specifically, 183, 183, 144, 133, 142, 134, and 114 volatile compounds were detected in samples F0, F1, F2, F3, F4, F5, and F6, respectively (Figure 3B). As the scenting time was prolonged, the number of volatile compounds identified in JYT exhibited a declining trend. However, the overall concentration of volatile components within the sample demonstrated an increasing trend.

The total volatile contents and component types are shown in Figure 3C. Compared with the tea base, JYT contained a significantly higher total amount of volatiles, following a characteristic “increase–then–decrease” trend with a prolonged scenting time. When the scenting time exceeded 14 h, a decline in total volatile content was observed. Prolonged scenting may lead to heat accumulation within the tea–flower pile due to continuous respiration of jasmine flowers, which could raise the local temperature. A potential increase in temperature may facilitate the volatilization of low-boiling constituents and reduce the tea’s moisture content, which in turn could impair the adsorption efficiency of the tea base [28]. However, because real-time measurements of pile temperature and humidity were not recorded in the present study, this proposed mechanism remains hypothetical and requires direct empirical validation.

### 3.3. Effect of Scenting on Volatile Components of Yellow Tea

**Figure 4:**
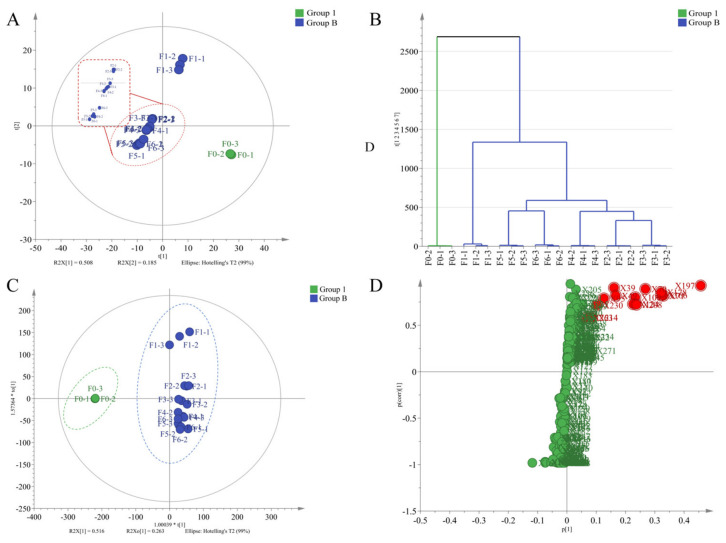
(**A**) Analysis of volatile compounds of yellow tea and JYT by PCA. (**B**) HCA. (**C**) OPLS-DA score plot of the JYT in group 1 and group B. (**D**) Compounds with VIPpred > 1 in samples. Notes: Figures (**A**–**C**), green (Group 1) denotes yellow tea, while blue (Group B) denotes JYT.

Principal Component Analysis (Figure 4A) demonstrated a sequential distribution of samples along the PC1 axis with increasing scenting time (0–16 h), revealing a strong correlation between volatile composition and scenting time. Samples F0 and F1 were located on the same side of the Y-axis, indicating minimal compositional differences between the tea base and the sample scented for 4 h, suggesting that shorter scenting time results in insufficient aroma adsorption. In contrast, F0, F1 and samples with ≥8 h of scenting were located on either side of the Y-axis, indicating substantial differences in volatile profiles after extended scenting.

HCA (Figure 4B) divided all samples into two major clusters with four hierarchical branches. Cluster 1 included the tea base (F0), whereas Cluster 2 comprised scented samples (≥4 h). Within Cluster 2, three subgroups were identified: Group 2 (F1, 4 h), Group 3 (F5 and F6, 14–16 h), and Group 4 (F2–F4, 8–12 h). This classification clearly demonstrates that scenting time significantly influences the volatile composition of JYT, consistent with sensory evaluation trends.

To further explore the changes in volatile components of yellow tea before and after scenting, an OPLS-DA model (Figure 4C) was established, with R^2^Y = 0.979 and Q^2^ = 0.971. The yellow tea sample (F0) and JYT samples (F1–F6) were distributed on both sides of the ordinate, indicating that the difference between the two groups was obvious. The result of the 200-time permutation test of the model, R^2^ = 0.138, Q^2^ = −0.581. Collectively, these results demonstrate that the OPLS-DA model is suitable for the discrimination and comparative analysis of aroma components in yellow tea before and after scenting.

Following the selection criteria proposed by An [29], where VIPpred > 1.0 (VIPpred values were derived from OPLS-DA), |log_2_FC| > 1.0, and *p* < 0.05, 14 key differential volatile compounds were identified: 8 esters, 2 alcohols, 3 hydrocarbons, and 1 heterocyclic compound (details listed in Table 1). These compounds were considered to play major roles in defining the aroma characteristics of JYT (Figure 4D).

### 3.4. The Key Characteristic Volatile Compounds in JYT

To determine the characteristic aroma components of JYT, the odor activity values (OAVs) of the identified volatile compounds were calculated (Table 2). Notably, all 14 compounds exhibited OAVs ≥ 1, indicating that these volatile components contribute significantly to the characteristic aroma profile of JYT and, thus, can be defined as its key characteristic aroma components.

**Figure 5:**
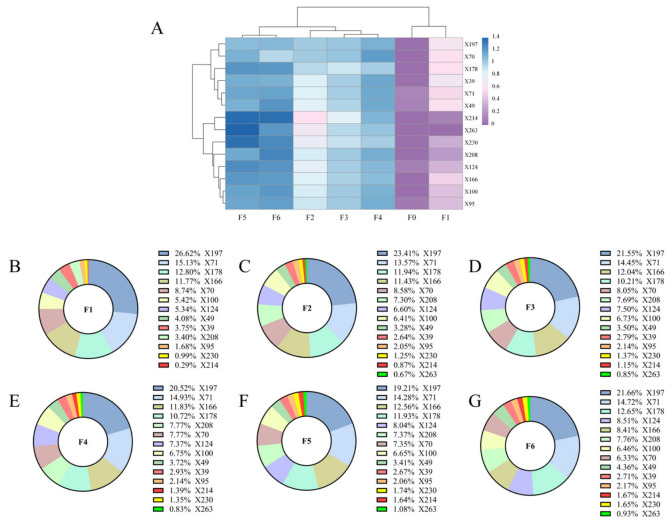
(**A**) Variation in the content of differential volatile compounds in different samples. (**B**–**G**) The proportion changes of 14 key aroma components in samples F1–F6.

A correlation heat map (Figure 5A) was constructed based on the 14 identified key volatile compounds, which revealed that the concentrations of the major differential volatile constituents were relatively low before the scenting process. Meanwhile, it was observed that the contents of these differential volatile compounds increased with prolonged scenting time, indicating that the scenting process exerts a pronounced influence on the accumulation of volatile components in JYT. Additionally, the concentration of (Z)-hex-3-en-1-yl (Z)-hex-3-enoate (X214) in samples F5 and F6 was notably higher compared to the other samples. From this observation, it is proposed that this compound may be closely associated with the distinctive dull odor of JYT. With the extension of the scenting time, the concentration of benzyl acetate (X197), initially constituting the largest proportion, exhibited a pattern characterized by an initial decrease followed by an increase. Concurrently, the content of methyl benzoate, known to impart floral and fruity aromas, gradually declined. However, the concentration proportion of methyl anthranilate, which contributes a floral aroma, increased by a factor of 2.15 (Figure 5B–G). The findings demonstrate that the aromatic quality of JYT is not dictated by the aroma concentration of an individual compound but arises from the harmonious coordination of a diverse array of compounds.

It is noteworthy that several major volatile compounds in JYT were also abundant in jasmine, including methyl benzoate, linalool, *α*-farnesene, and cis-3-hexenyl acetate, implying that these volatiles were absorbed from jasmine during scenting [32]. Prior studies have shown that benzyl acetate, *α*-farnesene, methyl benzoate, linalool, methyl anthranilate, and indole are typical floral aroma contributors [33,34], among which benzyl acetate and indole impart distinct jasmine notes. Methyl salicylate adds minty characteristics, being common in numerous florals [35], while cis-3-hexenyl acetate and *α*-farnesene provide grassy nuances [36]. Together, these compounds harmonize to form the unique aroma profile of JYT.

### 3.5. Volatile Compounds Coordinate Aroma Quality

**Figure 6:**
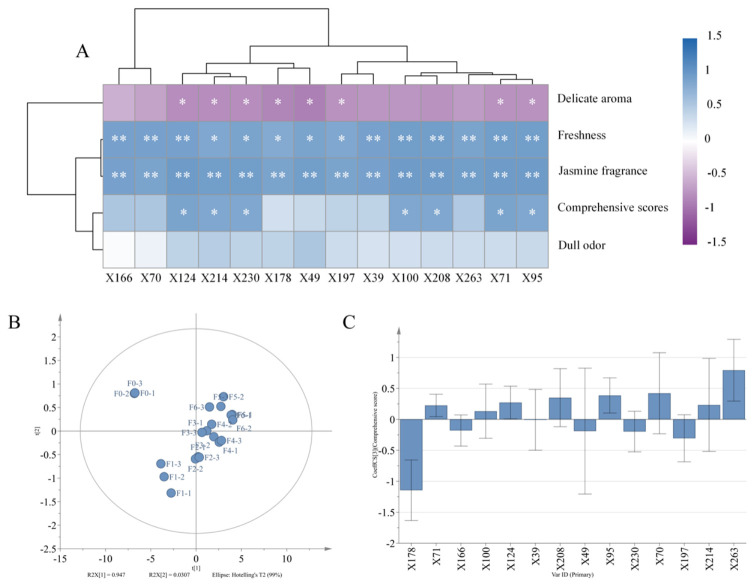
(**A**) Correlation between volatile compounds and JYT’s aroma. (**B**) Correlation coefficient diagram of volatile compounds and aroma comprehensive score. (**C**) PLS plots of different scenting times. Notes: The symbol “*” indicates a significant difference (*p* < 0.05), whereas “**” denotes a highly significant difference (*p* < 0.01).

To investigate the compounds primarily responsible for JYT aroma quality, a correlation analysis heatmap (Figure 6A) was constructed, combining sensory evaluation data and the concentrations of 14 major volatiles. The analysis revealed strong positive correlations between these volatiles and the jasmine aroma and freshness attributes. Among them, indole (X124), (Z)-hex-3-en-1-yl (Z)-hex-3-enoate (X214), *δ*-Cadinene (X230), methyl salicylate (X100), methyl anthranilate (X208), linalool (X71), and cis-3-hexenyl butyrate (X95) exhibited significant effects on the overall aroma score of jasmine tea, indicating their pivotal role in determining fragrance intensity. Interestingly, except for cis-3-hexenyl benzoate (X166), methyl benzoate (X70), cis-3-hexenyl acetate (X39), methyl salicylate (X100), methyl anthranilate (X208), and valencen (X263), all other key compounds showed significant negative correlations with the delicate aroma attribute, possibly due to the dominant jasmine fragrance masking the inherent yellow tea bases.

The concentrations of differential volatile compounds showed a continuous upward trend as scenting time increased. Based on the quantitative scores of these 14 volatiles and overall aroma quality (Figure 6B,C), PLS analysis was performed, R^2^Y = 0.88, Q^2^ = 0.809. Along the t_1_ axis, sample distribution followed the progression of scenting time, consistent with PCA results. The resulting regression equation illustrating the quantitative relationship between volatile compounds (X) and aroma quality (Y) is expressed as:

Y = −0.083 X178 + 0.019 X71 − 0.005 X166 + 0.007 X100 + 0.005 X124 + 0.008 X39 + 0.022 X208 − 0.007 X49 + 0.018 X95 − 0.011 X230 + 0.058 X70 + 0.022 X197 − 0.01 X214 + 0.012 X263.

Using this model, it was determined that JYT exhibited the best aroma quality when scented for 8–12 h, in accordance with the sensory evaluation results. To validate the model, additional JYT samples were produced following the procedure in Section 2.1 in August 2024 and assessed using this equation (Table S1). The predicted Y values corresponded strongly with sensory scores, displaying identical trends in variation. These findings support the applicability of the proposed formula for evaluating JYT aroma quality in the Hengzhou region.

## 4. Discussion

The 14 key differential volatiles in JYT are mostly derived from jasmine flowers and are mainly formed through adsorption during the scenting process. The content of *α*-farnesene is significantly increased after scenting (Table S2). It is biosynthesized via the “MVA pathway”: IPP and DMAPP are catalyzed by “FPPS” to form FPP, which is further converted into *α*-farnesene by “*α*-farnesene synthase (TPS)”. The related TPS genes are expanded and highly expressed in full-bloom flowers [37]. Indole is mainly derived from the tryptophan biosynthetic pathway. It is produced from indole-3-glycerol phosphate (IGP), catalyzed by tryptophan synthase *α* subunit (TSA) and acts as a key floral volatile in jasmine tea. During the scenting process, indole is predominantly released from jasmine flowers and enriched in tea leaves through adsorption [38]. Methyl salicylate and methyl benzoate are formed by O-methylation of salicylic and benzoic acids, respectively, and accumulate progressively [37]. *δ*-Cadinene and valencene, as typical sesquiterpenes, are biosynthesized from farnesyl diphosphate (FPP) under the catalysis of corresponding sesquiterpene synthases [39]. In addition, Benzyl acetate and benzyl alcohol are typical jasmine constituents [37].

In summary, most key compounds are jasmine-derived, and their accumulation kinetics are governed by floral emission rates, tea matrix adsorption, and post-scenting drying conditions. The total volatile content exhibited a trend of rising first and then declining during the scenting process. In the initial stage, jasmine flowers released abundant volatiles, and the yellow tea base showed a rapid physical adsorption rate that exceeded the rate of volatile dissipation. During the peak phases, adsorption approached saturation, the aroma-release rate of the flowers slowed, and the system reached dynamic equilibrium. In the subsequent declining phase, prolonged scenting caused the accumulation of metabolic heat and carbon dioxide, elevating the pile temperature. This accelerated the volatilization of low-boiling compounds and promoted the oxidative degradation of terpenes and esters. Meanwhile, the formation of a dull odour has a negative impact on the aroma quality of JYT. The number of detected volatiles decreased markedly, confirming that major characteristic compounds such as methyl anthranilate remained relatively stable, whereas minor or thermally unstable constituents were gradually lost.

Several limitations should be acknowledged. First, a single internal standard (ethyl decanoate) was used to quantify over 300 volatile compounds across diverse chemical classes. Different classes exhibit varying response factors, so the reported concentrations are semi-quantitative, suitable for relative comparison but not absolute quantification. Second, although seven trained panelists performed sensory evaluation, no inter-rater reliability metrics were calculated, limiting transparency of the consensus. Third, aroma-active compounds were identified by OAV rather than direct GC-olfactometry (GC-O), which could be affected by matrix effects. Future studies should address these limitations by using multiple internal standards, reporting reliability statistics, incorporating GC-O analysis, and testing model robustness across diverse conditions.

## 5. Conclusions

This study investigated the influence of scenting time on the aroma development of JYT by integrating instrumental analysis, sensory evaluation, and multivariate statistical techniques. Sensory evaluation indicated that the optimal scenting period is between 8 and 12 h, with the highest comprehensive aroma score at 10 h. A total of 300 volatile compounds were identified, and 14 key differential volatiles were verified as the primary contributors to the characteristic aroma profile of JYT. The PLS regression model established in this study effectively quantified the relationship between volatile compound concentrations and aroma comprehensive scores, and validation results confirmed its robustness for evaluating JYT aroma quality. The determination of this optimal scenting time (8–12 h), thus, provides a theoretical basis for refining and optimizing industrial processing protocols for JYT. Overall, this work offers technical guidance for the standardized production of high-quality JYT. Future research should deepen the understanding of aroma formation mechanisms during the scenting process and concurrently optimize multiple scenting parameters (e.g., temperature, humidity, and flower quantity), verify the generalizability of the aroma quality prediction model across different regions and raw materials, and elucidate the interactions between volatile and non-volatile components to more comprehensively improve the flavor profile of JYT.

## Figures and Tables

**Table 1:** Key volatile compounds (VIP > 1.0) of JYT.

Number	Component	VIP Pre	*p*-Value	Fragrance
X197	Benzyl acetate	7.92	*p* < 0.001	Jasmine fragrance ^a^
X71	Linalool	5.79	*p* < 0.001	Floral, woody, fruity ^a^
X166	cis-3-Hexenyl benzoate	5.68	*p* < 0.001	
X178	*α*-Farnesene	5.59	*p* < 0.001	Fruity, grassy ^a^
X70	Methyl benzoate	4.63	*p* < 0.001	Floral, fruity ^a^
X208	Methyl anthranilate	4.12	*p* < 0.001	Floral, fruity ^b^
X100	Methyl salicylate	4.09	*p* < 0.001	Fruity, minty ^b^
X124	Indole	3.98	*p* < 0.001	Jasmine fragrance ^a^
X49	Benzyl alcohol	2.94	*p* < 0.001	Faint sweet, floral ^a^
X39	cis-3-Hexenyl acetate	2.84	*p* < 0.001	Grassy, fruity ^b^
X95	cis-3-Hexenyl butyrate	2.20	*p* < 0.001	Fruity, green ^a^
X230	*δ*-Cadinene	1.82	*p* < 0.001	
X214	(Z)-hex-3-en-1-yl (Z)-hex-3-enoate	1.56	*p* < 0.001	Herbal, woody ^b^
X263	Valencen	1.22	*p* < 0.001	Fruity ^a^

All the aroma properties were obtained from ^a^ = Liu et al., 2023 [30] and ^b^ = Zheng et al., 2024 [31].

**Table 2:** The OAVs of characteristic aroma components in samples.

Number	Component	OAV	
		1	2
X197	Benzyl acetate	-	19.80
X71	Linalool	43.61	357.75
X166	cis-3-Hexenyl benzoate	0.11	25.32
X178	*α*-Farnesene	0.01	6.38
X70	Methyl benzoate	-	16.21
X208	Methyl anthranilate	55.94	613.58
X100	Methyl salicylate	0.45	40.15
X124	Indole	0.10	13.59
X49	Benzyl alcohol	0.25	2.29
X39	cis-3-Hexenyl acetate	1.16	77.95
X95	cis-3-Hexenyl butyrate	65.56	1025.64
X230	*δ*-Cadinene	-	210.08
X214	(Z)-hex-3-en-1-yl (Z)-hex-3-enoate	-	356.61
X263	Valencen	-	1.99

Notes: “1” represents F0; “2” represents F1–F6; and “-“ indicates that OAV cannot be calculated.

## Data Availability

The original contributions presented in this study are included in the article. Further inquiries can be directed to the corresponding authors.

## Supplementary Materials

The following supporting information can be downloaded at https://www.mdpi.com/article/10.3390/foods15101712/s1. Table S1: The key aroma components and corresponding contents of JYT samples. Table S2: The content of characteristic aroma components in samples.
