# Supplementary material for: Influence of Scenting Time on the Volatile Compounds and Sensory Attributes of Jasmine Yellow Tea
Source: Foods. 2026 May 13;15(10):1712. doi: 10.3390/foods15101712 (PMC13205161; doi:10.3390/foods15101712)
Supplement: Supplementary file 1 [file foods-15-01712-s001.zip › foods-4262215-supplementary.pdf]

Table S1. The key aroma components and corresponding contents of JYT samples

Component	CAS	RI[a]	RI[b]	Content(mg/kg)		
				Sample 1	Sample 2	Sample 3
α-Farnesene	502-61-4	1512	1508	2.97±0.50	3.10±0.34	4.79±0.35
Linalool	78-70-6	1103	1099	4.38±0.10	4.40±0.43	6.58±0.78
cis-3-Hexenyl benzoate	25152-85-6	1570	1570	2.46±0.41	3.12±0.46	4.26±0.96
Methyl salicylate	119-36-8	1201	1192	1.63±0.28	1.90±0.24	2.94±0.42
Indole	120-72-9	1299	1295	1.80±0.32	2.19±0.25	3.76±0.49
cis-3-Hexenyl acetate	3681-71-8	1009	1006	0.75±0.01	0.78±0.04	1.35±0.18
Methyl anthranilate	134-20-3	1348	1343	1.95±0.24	2.12±0.34	3.42±0.44
Benzyl alcohol	100-51-6	1037	1036	0.65±0.07	1.03±0.22	1.23±0.27
cis-3-Hexenyl butyrate	16491-36-4	1187	1187	0.69±0.06	0.65±0.05	0.63±0.04
δ-Cadinene	483-76-1	1523	1524	0.20±0.02	0.43±0.03	0.55±0.09
Methyl benzoate	93-58-3	1099	1094	2.12±0.32	2.34±0.33	2.57±0.84
Benzyl acetate	140-11-4	1169	1164	5.12±0.80	5.26±0.77	6.49±0.68
(Z)-hex-3-en-1-yl (Z)-hex-3-enoate	61444-38-0	1388	1389	0.39±0.05	0.42±0.05	0.78±0.14
Valencen	4630-07-3	1493	1492	0.18±0.02	0.00±0.00	0.00±0.00

Notes:

a indicates the retention index of n-alkanes on the DB-17MS column.

b retention indices retrieved from National Institute of Standards and Technology (NIST).

Table S2. The content of characteristic aroma components in the samples

Number	Component	Content (mg/kg)						
		F0	F1	F2	F3	F4	F5	F6
X197	Benzyl acetate	0.00±0.00	3.46±0.58	5.41±0.16	5.46±0.16	5.74±0.22	5.72±0.50	6.29±0.07
X71	Linalool	0.44±0.00	1.97±0.31	3.14±0.12	3.66±0.32	4.17±0.30	4.25±0.27	4.27±0.50
X166	cis-3-Hexenyl benzoate	0.01±0.00	1.53±0.30	2.64±0.07	3.05±0.26	3.31±0.37	3.74±0.39	2.44±0.25
X178	α-Farnesene	0.01±0.00	1.66±0.32	2.76±0.13	2.59±0.23	3.00±0.17	3.55±0.39	3.67±0.29
X70	Methyl benzoate	0.00±0.00	0.44±0.03	1.69±0.09	1.95±0.19	2.17±0.28	2.20±0.23	2.25±0.20
X208	Methyl anthranilate	0.16±0.01	0.69±0.11	1.53±0.02	1.90±0.14	2.06±0.06	2.39±0.34	2.47±0.19
X100	Methyl salicylate	0.02±0.00	0.71±0.10	1.48±0.08	1.70±0.23	1.89±0.10	1.98±0.10	1.87±0.23
X124	Indole	0.01±0.00	1.14±0.15	1.98±0.08	2.04±0.14	2.23±0.08	2.19±0.10	1.84±0.15
X49	Benzyl alcohol	0.10±0.00	0.53±0.06	0.76±0.01	0.89±0.14	1.04±0.08	1.02±0.10	1.26±0.08
X39	cis-3-Hexenyl acetate	0.01±0.00	0.49±0.08	0.61±0.03	0.71±0.04	0.82±0.08	0.80±0.05	0.79±0.06
X95	cis-3-Hexenyl butyrate	0.03±0.00	0.29±0.03	0.47±0.02	0.54±0.04	0.60±0.04	0.61±0.02	0.63±0.05
X230	δ-Cadinene	0.00±0.00	0.04±0.01	0.20±0.02	0.29±0.02	0.39±0.01	0.49±0.02	0.48±0.07
X214	(Z)-hex-3-en-1-yl (Z)-hex-3-enoate	0.00±0.00	0.13±0.03	0.29±0.02	0.35±0.02	0.38±0.01	0.52±0.02	0.48±0.04
X263	Valencen	0.00±0.00	0.00±0.00	0.15±0.02	0.21±0.01	0.23±0.01	0.32±0.00	0.27±0.02
